# Respiratory mechanics and gas exchanges in the early course of COVID-19 ARDS: a hypothesis-generating study

**DOI:** 10.1186/s13613-020-00716-1

**Published:** 2020-07-16

**Authors:** J.-L. Diehl, N. Peron, R. Chocron, B. Debuc, E. Guerot, C. Hauw-Berlemont, B. Hermann, J. L. Augy, R. Younan, A. Novara, J. Langlais, L. Khider, N. Gendron, G. Goudot, J.-F. Fagon, T. Mirault, D. M. Smadja

**Affiliations:** 1 Université de Paris, Innovative Therapies in Haemostasis, INSERM, 75006 Paris, France; 2 Intensive Care Unit and Biosurgical Research Lab (Carpentier Foundation), AH-HP, Georges Pompidou European Hospital, 20 Rue Leblanc, 75015 Paris, France; 3 Intensive Care Unit, AH-HP, Georges Pompidou European Hospital, Université de Paris, 75015 Paris, France; 4 Université de Paris, PARCC, INSERM, 75015 Paris, France; 5 Emergency Department, AP–HP, Georges Pompidou European Hospital, 75015 Paris, France; 6 Plastic Surgery Department, AP-HP, Georges Pompidou European Hospital, Université de Paris, 75015 Paris, France; 7 Vascular Medicine Department and Biosurgical Research Lab (Carpentier Foundation), AP-HP, Georges Pompidou European Hospital, Université de Paris, 75015 Paris, France; 8 Hematology Department and Biosurgical Research Lab (Carpentier Foundation), AH-HP, Georges Pompidou European Hospital, 75015 Paris, France; 9 Vascular Medicine Department, AP-HP, Georges Pompidou European Hospital, 75015 Paris, France

**Keywords:** ARDS, COVID-19, Physiological dead-space, Ventilatory ratio

## Abstract

**Rationale:**

COVID-19 ARDS could differ from typical forms of the syndrome.

**Objective:**

Pulmonary microvascular injury and thrombosis are increasingly reported as constitutive features of COVID-19 respiratory failure. Our aim was to study pulmonary mechanics and gas exchanges in COVID-2019 ARDS patients studied early after initiating protective invasive mechanical ventilation, seeking after corresponding pathophysiological and biological characteristics.

**Methods:**

Between March 22 and March 30, 2020 respiratory mechanics, gas exchanges, circulating endothelial cells (CEC) as markers of endothelial damage, and D-dimers were studied in 22 moderate-to-severe COVID-19 ARDS patients, 1 [1–4] day after intubation (median [IQR]).

**Measurements and main results:**

Thirteen moderate and 9 severe COVID-19 ARDS patients were studied after initiation of high PEEP protective mechanical ventilation. We observed moderately decreased respiratory system compliance: 39.5 [33.1–44.7] mL/cmH_2_O and end-expiratory lung volume: 2100 [1721–2434] mL. Gas exchanges were characterized by hypercapnia 55 [44–62] mmHg, high physiological dead-space (*V*_D_/*V*_T_): 75 [69–85.5] % and ventilatory ratio (VR): 2.9 [2.2–3.4]. *V*_D_/*V*_T_ and VR were significantly correlated: *r*^2^ = 0.24, *p* = 0.014. No pulmonary embolism was suspected at the time of measurements. CECs and D-dimers were elevated as compared to normal values: 24 [12–46] cells per mL and 1483 [999–2217] ng/mL, respectively.

**Conclusions:**

We observed early in the course of COVID-19 ARDS high *V*_D_/*V*_T_ in association with biological markers of endothelial damage and thrombosis. High *V*_D_/*V*_T_ can be explained by high PEEP settings and added instrumental dead space, with a possible associated role of COVID-19-triggered pulmonary microvascular endothelial damage and microthrombotic process.

## Introduction

Patients with Covid-19 pneumonia fulfilling Berlin criteria of ARDS may present some specific features, as compared to typical forms of the syndrome [1]. Such reported features are severe hypoxemia contrasting with relative preservation in respiratory mechanics [1, 2], and a common hypercapnia with high ventilatory ratio (VR) [2]. Low recruitability, improved by body positioning, has been reported by some authors [3], while others mentioned that most of the patients were highly recruitable [4]. Mauri et al. reported in 10 intubated COVID-19 ARDS patients a large variability in potential for lung recruitment together with an elevated dead space fraction evaluated by electrical impedance tomography (EIT) [5].

During the initial epidemic in China, abnormal coagulation profiles were observed in severe COVID-19 patients. D-dimers above 1000 ng/mL was an independent risk factor of in-hospital death [6]. In another study, D-dimers were also correlated with mortality [7]. The hypothesis of microthrombosis was proposed since high levels of creatinine were associated with higher levels of D-dimers (> 500 ng/mL), in favor of a microthrombotic origin for kidney failure [8].

Endothelial damage at the microvascular level may thus play an important role not only in the incidence of renal failure, but also for some aspects of the pathogenesis of respiratory failure, beside alveolar insults. Indeed, the SARS-CoV-2 receptor (ACE2) is strongly expressed in endothelial cells [9]. Since the lung accepts the whole of the cardiac output in its rich vascular and microvascular bed, it could be therefore possible that infection of endothelial cells could induce pulmonary endothelial lesions, triggering activation of coagulation. Accordingly, endothelial cells viral infection, pulmonary vascular endothelialitis and pulmonary vascular microthrombosis are increasingly reported in autopsy studies [10–12]. Finally, based on an initial series of 40 COVID-19 hospitalized patients and on an independent cohort of 32 COVID-19 patients, we recently reported the interest of angiopoietin-2, a marker of endothelial activation, for predicting the need for ICU admission [13].

Based on initial clinical findings observed in a Covid-19 ARDS patient in January 2020, we planned to systematically investigate respiratory mechanics and gas exchanges in Covid-19 ARDS patients subsequently admitted to the medical ICU of the Georges Pompidou European Hospital.

## Patients and methods

### Study design and population

A COVID-19 80-year-old patient without history of respiratory disease was placed on invasive mechanical ventilation (IMV) after non-invasive ventilation failure on Jan 27, 2020. After tracheal intubation, the patient rapidly fulfilled severe ARDS criteria. A high-PEEP protective ventilation strategy was used, as part of our respiratory bundle [14]. While the respiratory system compliance was 25 mL/cmH_2_O and the PaO_2_/FiO_2_ ratio 183 mmHg, the patient exhibited very high PaCO_2_: 103 mmHg and VR: 3.5; with the following settings: *V*_T_: 6 mL/Kg PBW, RR: 22/min, PEEP: 16 cmH_2_O, FiO_2_: 60%. Increasing respiratory rate from 22 to 35/min was associated with a decrease in PaCO_2_ from 103 to 79 mmHg. The patient was thereafter transferred to another hospital because of the absence of biosafety level 3 laboratory in our center [15]. Such a respiratory pattern prompted us to plan to further precisely analyze the respiratory characteristics of other COVID-19 ARDS patients admitted in the ICU.

Results were obtained thereafter between March 22, 2020 and March 30, 2020 in 22 consecutive COVID-19 ARDS patients without history of chronic respiratory disease. No patient was clinically suspected of pulmonary embolism at the time of measurements. A prophylactic anticoagulation regimen (enoxaparin 40 mg once daily subcutaneously) was administered. All patients were included in the French-COVID national cohort after informed consent of proxies or family members by phone, due to quarantine. Additionally, proxies or family members gave also an informed consent by phone for a formalized local process of collecting biological samples in relation to cardiovascular, metabolic or renal diseases (Comité de Protection des Personnes Ile-De-France II, IRB registration 00001072, approval: November 11, 2016).

While using a 5-cmH_2_O PEEP setting, 13 patients fulfilled the Berlin criteria for moderate ARDS and 9 patients for severe ARDS, with a median [IQR] PaO_2_/FiO_2_ value of 108 [87–134]. We therefore used a high-PEEP protective ventilation strategy, as part of our respiratory bundle in ARDS patients [14]. We present measurements obtained early during the IMV course in deeply sedated and paralyzed patients. The CareScape R860 ventilator (GE Healthcare, USA) was used, allowing the following measurements or calculations:

- Respiratory mechanics: plateau pressure (Pplateau), total PEEP (PEEPtot), driving pressure (DP), respiratory system compliance (Crs), end-expiratory lung volume (EELV) as determined by the nitrogen washin–washout method.
- Gas exchanges: arterial blood gases, end-tidal expired CO_2_ (E_T_CO_2_), PaO_2_/FiO_2_ ratio, alveolo-arterial O_2_ difference (DAaO_2_), VR, physiological dead space (*V*_D_/*V*_T_) as calculated by the respirator using the Enghoff–Bohr equation, O_2_ total body uptake (VO_2_), CO_2_ total body production (VCO_2_).

We also obtained additional *V*_D_/*V*_T_ measurements in patients in whom PEEP level was lowered of at least 5 cmH_2_O within 4 days following initial measurements, when clinically indicated.

### Laboratory confirmation of SARS-CoV-2 infection

Nasopharyngeal swabs were collected in universal transport medium (Xpert^®^ nasopharyngeal sample collection kit) at hospital admission. SARS-CoV-2 was detected using Allplex™ 2019-nCoV Assay (Seegene), a multiplex real-time PCR assay that detects three target genes (E gene, RdRP gene and N gene) in a single tube. Data were automatically analyzed using Seegene viewer software. Only qualitative data were available. Broncho-alveolar lavage procedures were performed for confirmation if necessary.

### Routine blood examinations

All samples were collected on sodium heparin and 0.129 M trisodium citrate tubes (9NC BD Vacutainer, Plymouth, UK) at the same time than respiratory measurements. Routine blood examinations were complete blood count, plasmatic biochemical tests including C-reactive protein (CRP) (upper normal limit: 5 mg/L). Coagulation parameters including fibrinogen (normal limits between 1.5 and 3.5 g/L) were explored with a STA-R^®^ Max (Stago) coagulometer. D-dimers concentrations (upper normal limit: 500 ng/mL) were determined using Vidas D-Dimer (BioMérieux) according to the manufacturer’s instructions.

### CECs quantification

Circulating endothelial cells (CECs) were quantified at the same time as respiratory measurements, with an upper normal limit of 10 CECs per mL of whole blood. Peripheral venous blood samples were collected on EDTA after having always discarded the first milliliter of blood to avoid presence of endothelial cells dislodged by puncture. CECs were isolated by immunomagnetic separation with mAb CD146-coated beads and stained with the fluorescent probe acridine orange as previously described [16–19].

### Statistical analysis

Descriptive statistics were used to summarize the data. Results are reported as medians and interquartile ranges and categorical variables were summarized as counts and percentages. The correlation of quantitative variables (between VR and *V*_D_/*V*_T_ and between VR and VCO_2_) were assessed using the Kendal rank correlation coefficient. All statistical analyses were performed using R software (Version 2019 R: A language and environment for statistical computing. R Foundation for Statistical Computing, Vienna, Austria).

## Results

Among the 22 COVID-19 ARDS patients, 19 (86.4%) were males with a median age of 65 [55–73] years. Simplified Acute Physiologic Score II (SAPSII) and Sequential Organ Failure Assessment (SOFA) scores were 55 [43–63] and 9 [7–11], respectively. Measurements were obtained 1 [1–4] day after intubation. Criteria for intubation followed French national guidelines [20]. Delay between first symptoms and tracheal intubation was 9 [7–11] days.

We observed a lymphopenia: 0.64 G/L [0.42–0.91]), an increase in CRP: 167 mg/L [105–209] and in fibrinogen: 6.4 g/L [5.15–6.90]. Patients had an increase in D-dimers: 1483 ng/mL [999–2217]. The proportions of patients with values of D-dimers above 500 ng/mL and 1000 ng/mL were 82% and 60%, respectively. PT ratio was in normal values (89% [79–99]) and none of the patients had positive fibrin monomers at ICU admission. In the context of COVID-19 with high levels of D-Dimers, CRP and fibrinogen at admission, the low level of fibrin monomers allowed us excluding a disseminated intravascular coagulation process. Patients had an increase in CECs: 24/mL [12–46].

**Fig. 1:**
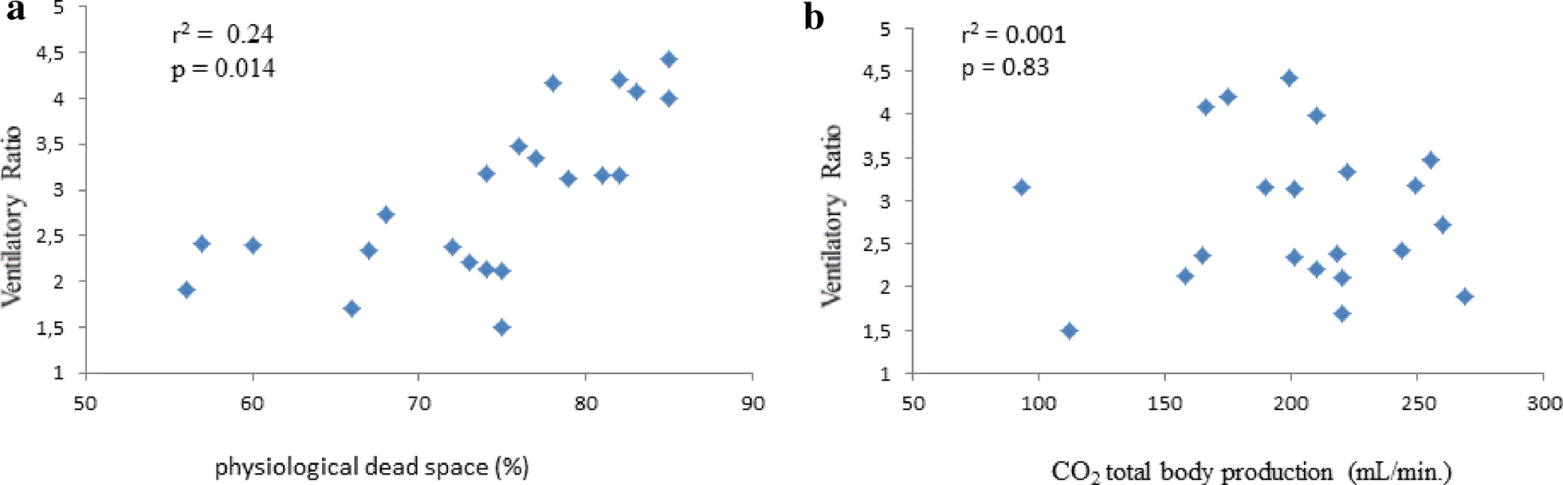
Correlations between different respiratory parameters. **a** Correlation between physiological dead space and ventilatory ratio in 22 COVID-19 ARDS patients studied early after intubation and initiation of protective ventilation. **b** Correlation between CO_2_ total body production and ventilatory ratio

Values for respiratory and hemodynamic parameters are reported in Table 1. Correlations between VR and *V*_D_/*V*_T_ and between VR and VCO_2_ (one lacking data) are illustrated in Fig. 1. Mainly, we observed a statistically significant positive correlation between VR and *V*_D_/*V*_T_ (*r*^2^ = 0.24, *p* = 0.014). No correlation was observed between VR and VCO_2_ (*r*^2^ = 0.001, *p* = 0.83).

**Table 1:** Respiratory and hemodynamic parameters observed early in the course of protective mechanical ventilation in 22 COVID-19 ARDS patients

Ventilator settings	
*V*_T_ set at 6 mL/kg PBW (mL)	412 [356–425]
RR (/min)	33 [28.5–35]
PEEP (cmH_2_O)	16 [15–17]
FiO_2_ (%)	45 [40–58]
Respiratory mechanics	
*P*_plateau_ (cmH_2_O)	27 [25–28]
PEEPtot (cmH_2_O)	16.5 [16–18]
DP (cmH_2_O)	9.5 [9–11.75]
Crs (mL/cmH_2_O)	39.5 [33.1–44.7]
EELV (mL)	2100 [1721–2434]
Gas exchanges	
PaCO_2_ (mmHg)	55 [44–62]
PaO_2_/FiO_2_	198 [167–298]
DAaO_2_ (mmHg)	136 [102–209]
E_T_CO_2_ (mmHg)	38 [33–45]
VR	2.9 [2.2–3.4]
*V*_D_/*V*_T_ (%)	75 [69–80.5]
VO_2_ (mL/min)	280 [226–327]
VCO_2_ (mL/min)	210 [175–222]
Hemodynamic support	
Catecholamine support *n* (%)	8 (36%)

*V*_*T*_ tidal volume, *PBW* predicted body weight, *RR* respiratory rate, *P*_*plateau*_ plateau pressure, *PEEP*_*tot*_ total PEEP measured during a prolonged expiratory pause, *DP* driving pressure, *Crs* respiratory system compliance, *EELV* end-expiratory lung volume, *DAaO*_*2*_ alveolo-arterial difference in O_2_ partial pressures, *E*_*T*_*CO*_*2*_ end-tidal expired CO_2_, *VR* ventilatory ratio, *V*_*D*_*/V*_*T*_ physiological dead space, *VO*_*2*_ O_2_ total body uptake, *VCO*_*2*_ CO_2_ total body production

*V*_D_/*V*_T_ measurements after reducing PEEP of at least 5 cmH_2_O were obtained in 7 patients, with a median delay between initial measurements and “low PEEP” of 2 [2–3.5] days. The median “high PEEP” value was of 16 [16–17.5] cmH_2_O and the median “low PEEP” value was: 11 [10–12] cmH_2_O, *p* = 0.02. Median *V*_D_/*V*_T_ values at high and low PEEP were 74 [66–82] % and 76 [71.5–80.5] %, respectively (*p* = 0.867). Similar measurements were not available in the 15 other patients, due to the course of the disease.

## Discussion

We present a series of 22 consecutive COVID-19 ARDS patients studied shortly after intubation and initiation of a high-PEEP protective ventilation strategy, combining physiological respiratory and biological results. To the best of our knowledge, our series is the first to report EELV and *V*_D_/*V*_T_ values in COVID-19 ARDS [21]. We observed mainly high values of *V*_D_/*V*_T_ and VR, in parallel with elevated markers of endothelial damage and thrombotic process. Accordingly, we suggest a pathophysiological contributing hypothesis in addition to the expected role of added instrumental dead space and high PEEP setting.

One main finding is the observation of a high physiological dead space, as compared to previously published series of non-COVID-19 ARDS patients summarized in Table 2 [22–31]. Based on VR measurements in 8 patients, Liu et al. hypothesized an increase in alveolar dead space leading to a decrease in alveolar ventilation favoring hypercapnia [2]. Based on EIT, Mauri et al. reported an elevated dead space fraction and suggested that it could be a specific pathophysiological trait [5]. Importantly, the percentage of dead space quantified by EIT differs from the traditional measure provided by capnography for two main reasons (*T. Mauri, personal communication*): EIT measures dead space inside the lungs, the contribution of the anatomical dead space being minimal and the EIT-based dead space being quantified as a percentage of the lung volume and not of the tidal volume. Accordingly, the median V_T_ in our series was only 20% of the median EELV value. Therefore, we believe that capnography measurements in the present series and EIT measurements by Mauri et al. are not contradictory but rather complementary, as previously suggested [21].

**Table 2:** Published reference values on *V*_D_/*V*_T_ (Enghoff) and VR in non-COVID-19 ARDS patients

First author	Year of publication	*n*	PEEP level (cmH_2_O)	*V*_D_/*V*_T_ (Enghoff) (%)	VR	Comments
Nuckton [22]	2002	179	8.5 ± 3	58 ± 10	NR	
Lucangelo [23]	2008	10	10 ± 3	54 ± 14	NR	
Fengmei [24]	2012	12 deceased 11 survived	6 6	64 ± 8 53 ± 4	NR	Fixed PEEP level
Kallet [25]	2014	99	10 ± 3	57 ± 11	NR	
Beitler [26]	2015	210	10 ± 4	60 ± 12	NR	
Sinha [27]	2018	520	11 ± 4	63 ± 12	1.9 ± 0.6	28 patients with mild ARDS
Cogniat [28]	2018	14	16	69 [59-77]	NR	7 patients with mild ARDS HME (internal volume: 40 mL) *V*_D_/*V*_T_ not influenced by PEEP level (0 to 16 cmH_2_O)
Van Meenen [29]	2019	41 deceased 49 survived	15 [11-16] 15 [10-16]	43 [34-51] 27 [22-36]	NR	
Morales-Quintero [31]	2019	288 deceased 652 survived	10 [6-13] 10 [5-12]	NR	1.8 [1.5-2.3] 1.6 [1.4-2]	
Ospina Tascon [30]	2020	42	12 [10-15]	54 [45-61]	NR	
Present study		22	16 [15-17]	75 [69-80.5]	2.9 [2.2-3.4]	COVID-19 patients

*V*_*D*_*/V*_*T*_ physiological dead space, *VR* ventilatory ratio, *HME* heat and moisture exchange filter

Unfortunately, we were not able to perform volumetric capnography. Therefore, it is not possible to distinguish between increases in anatomical and instrumental dead space and alveolar dead space. Accordingly, the increase in *V*_D_/*V*_T_ could be in relation with different factors:

1. Pulmonary embolism was clinically ruled out in all 22 patients at the time of measurements. Despite prophylactic anticoagulation, a pulmonary embolism was diagnosed on CT-scan during follow-up in 3 patients 24 h, 5 days and 15 days after measurements. Removing the first patient from the analysis did not modify the results of the study.
2. Alveolar overdistension with compression of intra-alveolar vessels could have occurred in some pulmonary territories in relation to the high-PEEP protective ventilation. However, no deterioration in Crs was reported after switching from conventional low-PEEP mechanical ventilation to high-PEEP protective ventilation. Also, plateau pressures were kept within a safe range and only 8 patients needed catecholamine support at the time of measurements. We did not observe a decrease in *V*_D_/*V*_T_ in 7 patients after decreasing PEEP during the first days after initial measurements. Finally, Guo and colleagues previously studied dead space fraction changes during PEEP titration in a series of 23 ARDS patients [24]. The absolute difference in mean *V*_D_/*V*_T_ at PEEP 16 and 6 cmH_2_O was less than 5%. Therefore, it could be possible that alveolar overdistension with compression of intra-alveolar vessels could only partially explain our results.
3. An increase in instrumental dead-space was also a contributor to high *V*_D_/*V*_T_ values. We used closed suction systems adding 4 mL to the instrumental dead space and heat and moisture exchange filters with 36 mL of internal volume. The metabolic sensor, placed at the Y-piece level, has an internal volume of 9.5 mL, but permits to eliminate the influence of compression/decompression phenomenon. Assuming an added instrumental dead space of 49.5 mL permits to calculate a median corrected physiological dead space of 0.63, remaining in a high range.
4. Finally, we can propose as an additional hypothesis the occurrence of a unusually diffuse microcirculatory dysfunction, as highlighted in a hypothesis-generating observational study in ARDS patients suggesting an inverse correlation between small vessels perfusion and *V*_D_/*V*_T_ [30]. Since the SARS-CoV-2 receptor (ACE2) is strongly expressed in endothelial cells [9], infection of endothelial cells could have induced pulmonary endothelial lesions and triggered activation of coagulation, at least in some patients. Accordingly, we found elevated values of CECs (as a marker of endothelial lesion) and of D-dimers (as thrombosis marker). However, we have to mention that increased CECs and D-dimers are not specific of COVID-19 ARDS as compared to septic ARDS of other etiologies: Moussa reported a median CEC count of 27.2 cells/mL in 17 moderate/severe ARDS patients and Helms reported median D-dimers levels of 2270 ng/mL and 3400 ng/mL in 150 COVID-19 and 145 matched non-COVID-19 ARDS patients, respectively [32, 33]. Moreover, pulmonary in situ thrombosis and endothelial damage were reported more than 30 years ago [34, 35]. One can speculate that COVID-19 ARDS could be characterized in the more severe patients by such a large pulmonary microvasculature dysfunction.

Ventilatory ratio is a simple bedside index of impaired efficiency of ventilation, but we found a weaker correlation between *V*_D_/*V*_T_ and VR as compared a reference study from Sinha, confirming their assumption that VR cannot be used to estimate *V*_D_/*V*_T_ [27]. Accordingly, we suggest to clinicians to be cautious about the index, preventing misuse or misinterpretation.

In line with previous publications, we found only moderately decreased compliance. Additionally, this was accompanied by moderately decrease EELV values as compared to normal values, contrasting with impairment in gas exchanges.

Our study suffers from limitations. Mainly the study is a monocentric one with patients studied only early in the course of the disease, without a formalized control group of non-COVID-19 ARDS patients. In addition, we cannot provide *V*_D_/*V*_T_ measurements at low PEEP level prior measurements at high PEEP levels, as well as measurements with reduction in the instrumental dead-space. Accordingly, it can only be considered as hypothesis generating, in line with studies from other groups [5, 21]. Confirmation of diffuse pulmonary microvascular damage and microthrombosis in a larger number of COVID-19 ARDS patients and by other groups would be the only way to confirm or not the hypothesis.

## Data Availability

The datasets used and/or analyzed during the current study are available from the corresponding author on reasonable request.
